# Correction to: A whole system approach to increasing children’s physical activity in a multi-ethnic UK city: a process evaluation protocol

**DOI:** 10.1186/s12889-022-12514-4

**Published:** 2022-01-11

**Authors:** Jennifer Hall, Daniel D. Bingham, Amanda Seims, Sufyan Abid Dogra, Jan Burkhardt, James Nobles, Jim McKenna, Maria Bryant, Sally E. Barber, Andy Daly-Smith

**Affiliations:** 1 Bradford Institute for Health Research, Bradford Teaching Hospitals NHS Foundation Trust, Bradford Royal Infirmary, Duckworth Lane, Bradford, BD9 6RJ UK; 2 Faculties of Life Sciences and Health Studies, University of Bradford, Richmond Road, Bradford, BD7 1DP UK; 3 The National Institute for Health Research Applied Research Collaboration West (NIHR ARC West), University Hospitals Bristol National Health Service Foundation Trust, Bristol, UK; 4 Population Health Sciences, Bristol Medical School, University of Bristol, Bristol, UK; 5 School of Sport, Carnegie, Leeds Beckett University, Leeds, LS6 3QT UK; 6 Department of Health Sciences, Seebohm Rowntree Building, University of York, Heslington, York, YO10 5DD UK; 7 The Hull York Medical School, University of York, York, YO10 5DD UK; 8 Centre for Applied Education Research, Wolfson Centre for Applied Health Research, Bradford Royal Infirmary, Bradford, West Yorkshire UK

**Correction to: BMC Public Health 21, 2296 (2021)**

**https://doi.org/10.1186/s12889-021-12255-w**

Following publication of the original article [1], the authors identified that the funding section was incomplete. The incorrect and correct funding section is shown below. The original article has been updated.

- The incorrect funding section is:
  - The authors’ involvement was supported by Sport England’s Local Delivery Pilot – Bradford; weblink: https://www.sportengland.org/campaigns-and-our-work/local-delivery and the National Institute for Health Research (NIHR) Yorkshire and Humber Applied Research Collaboration (ARC). Sport England is a non-departmental public body under the Department for Digital, Culture, Media and Sport (DCMS). James Nobles’ time was supported by the NIHR ARC West. The views expressed in this article are those of the author(s) and not necessarily those of Sport England, DCMS, NIHR or the Department of Health and Social Care.
- The correct funding section is:
  - The authors’ involvement was supported by Sport England’s Local Delivery Pilot – Bradford; weblink: https://www.sportengland.org/campaigns-and-our-work/local-delivery and the National Institute for Health Research (NIHR) Yorkshire and Humber Applied Research Collaboration (ARC). Sport England is a non-departmental public body under the Department for Digital, Culture, Media and Sport (DCMS). James Nobles’ time was supported by the NIHR ARC West. The views expressed in this article are those of the author(s) and not necessarily those of Sport England, DCMS, NIHR or the Department of Health and Social Care.
    Sally Barbers and Maria Bryants’ time was supported by the UK Prevention Research Partnership (MR/S037527/1), an initiative funded by UK Research and Innovation Councils, the Department of Health and Social Care (England) and the UK devolved administrations, and leading health research charities. Weblink: https://mrc.ukri.org/research/initiatives/prevention-research/ukprp/
