# Supplementary figures and images for: A whole system approach to increasing children’s physical activity in a multi-ethnic UK city: a process evaluation protocol
Source: BMC Public Health. 2021 Dec 18;21:2296. doi: 10.1186/s12889-021-12255-w (PMC8684063; doi:10.1186/s12889-021-12255-w)

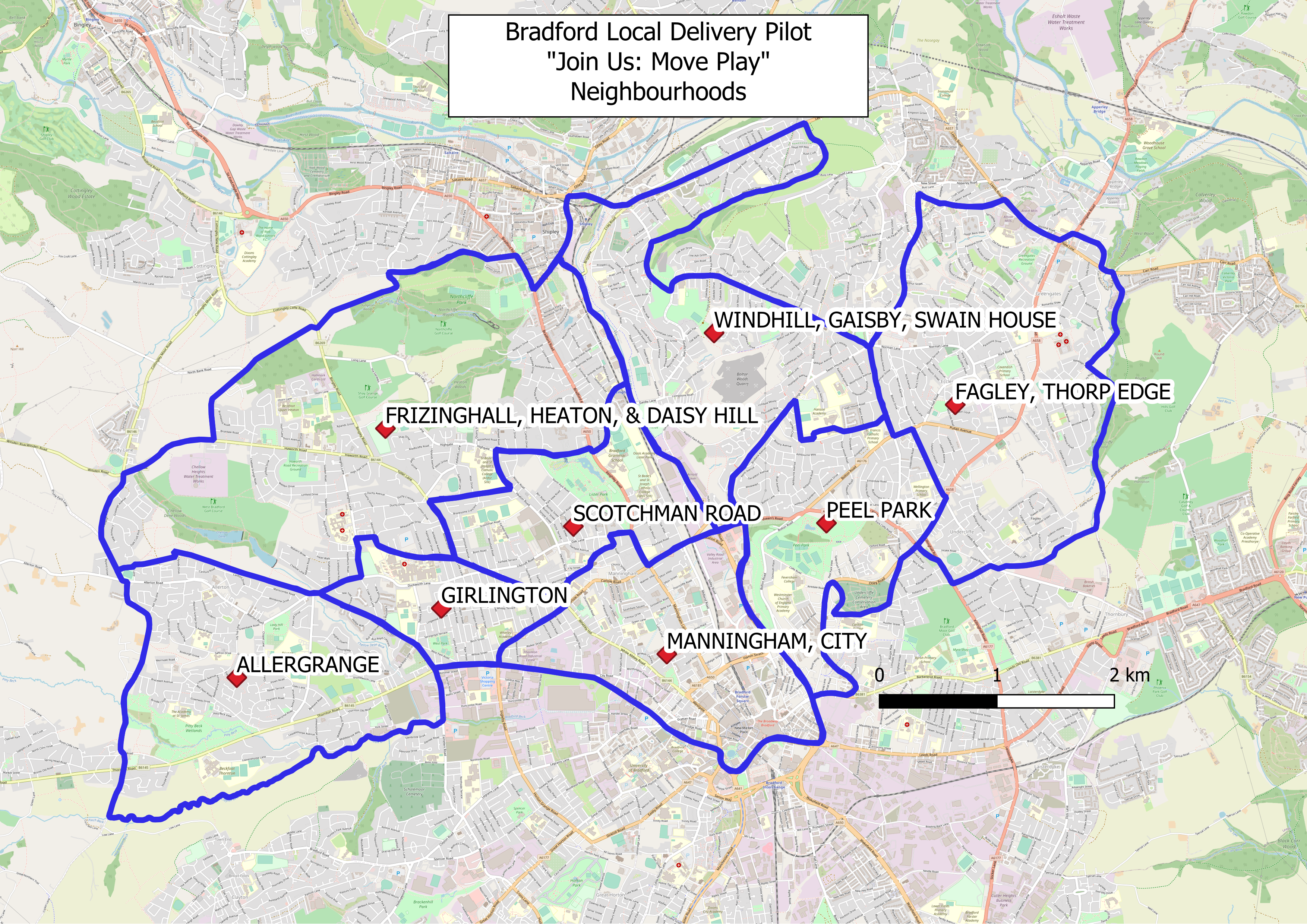

Supplement: Supplementary file 1 — Additional file 1. JU:MP neighbourhood map. A geographical map of the JU:MP neighbourhood boundaries within the LDP area. File extension: png. The map depicted in Additional file 1 is adapted from QGIS Geographic Information System (2021). Open Source Geospatial Foundation Project. http://ggis.osgeo.org. [file 12889_2021_12255_MOESM1_ESM.png]
